# How nearby nutrients shape tumor growth

**DOI:** 10.7554/eLife.89825

**Published:** 2023-07-17

**Authors:** Nada Kalaany

**Affiliations:** 1 Division of Endocrinology, Boston Children's Hospital, Harvard Medical School Boston United States; 2 Broad Institute of MIT and Harvard Cambridge United States

**Keywords:** metabolism, tumor microenvironment, amino acid homeostasis, cancer, nutrient stress, immunotherapy, Human, Mouse

## Abstract

Studying the nutrient composition immediately surrounding pancreatic cancer cells provides new insights into their metabolic properties and how they can evade the immune system to promote disease progression.

**Related research article** Apiz Saab JJ, Dzierozynski LN, Jonker PB, AminiTabrizi R, Shah H, Menjivar RE, Scott AJ, Nwosu ZC, Zhu Z, Chen RN, Oh M, Sheehan C, Wahl DR, Pasca di Magliano M, Lyssiotis CA, Macleod KF, Weber CR, Muir A. 2023. Pancreatic tumors exhibit myeloid-driven amino acid stress and upregulate arginine biosynthesis. *eLife* **12**:e81289. doi: 10.7554/eLife.81289.

**Related research article** Menjivar RE, Nwosu ZC, Du W, Donahue KL, Hong HS, Espinoza C, Brown K, Velez-Delgado A, Yan W, Lima F, Bischoff A, Kadiyala P, Salas-Escabillas D, Crawford HC, Bednar F, Carpenter E, Zhang Y, Halbrook CJ, Lyssiotis CA, Pasca di Magliano M. 2023. Arginase 1 is a key driver of immune suppression in pancreatic cancer. *eLife* **12**:e80721. doi: 10.7554/eLife.80721.

Changes in the way that tumor cells use nutrients to produce energy is a hallmark of cancer (Hanahan and Weinberg, 2011). Yet, the relentless search for metabolic features specific to cancer cells, which could then become therapeutic targets, has faced many challenges (Martínez-Reyes and Chandel, 2021) – including concerns around toxicity and the ability for these cells to easily alter their metabolism to resist therapy.

The fact that the metabolism of lab-grown tumor cells may differ from those growing inside the body has also presented a serious hurdle. What food type is available in the immediate surrounding, or ‘microenvironment’, of a cell can have an immense impact on which nutrients it takes up and metabolizes, and how. Therefore, studying isolated tumor cells exposed to nutrient levels that do not match what they normally encounter in their microenvironment could lead to misleading results that hinder how scientists can both understand and treat cancer.

Some researchers have attempted to optimize the media cancer cells are cultured in so that it mimics the composition of nutrients circulating in human blood (Cantor et al., 2017; Vande Voorde et al., 2019). In 2019, a group – led by Matthew Vander Heiden and Alexander Muir – decided to take this one step further and profiled the metabolic content in the interstitial fluid immediately surrounding tumor cells in mice genetically modified to have pancreatic ductal adenocarcinoma (PDA), the most common form of pancreatic cancer (Sullivan et al., 2019). This revealed that some nutrients are present at different levels than in blood. Now, in eLife, two teams in the United States – one led by Alexander Muir with Juan Apiz-Saab and Lindsey Dzierozynski as co-first authors, and one led by Costas Lyssiotis and Marina Pasca di Magliano with Rosa Menjivar as first author – report how the nutrients available in the microenvironment of pancreatic tumors shape the metabolism of cancer cells and protect their growth (Apiz Saab et al., 2023; Menjivar et al., 2023).

Apiz-Saab, Dzierozynski et al. (who are based at the University of Chicago and University of Michigan) used the nutrient composition of the interstitial fluid of PDA tumors as a template to generate a customized medium for cancer cells named TFIM (short for Tumor Interstitial Fluid Medium). The medium contained plenty of glutamine, an amino acid preferred by tumors which is also abundant in blood, but was significantly depleted in arginine compared to blood plasma. While some differences remained (for instance, tumors in vivo contain a greater range of cell types), PDA cells cultured in TIFM adopted a cellular state similar to that observed in tumors growing inside a host.

Further investigations revealed that TIFM-grown cells were able to activate the urea cycle, a series of biochemical reactions that normally occur in the liver. This metabolic process allowed the cancer cells to produce their own arginine, which may explain why PDA cells are able to survive in a tumor microenvironment lacking this amino acid (Figure 1A). The cells relied on the presence of the intermediary molecule citrulline, which is present in TIFM but absent in standard cancer cell media, to be able to complete the urea cycle. This result was further supported by in vivo experiments conducted in mice bearing PDA tumors in their pancreas.

**Figure 1.**
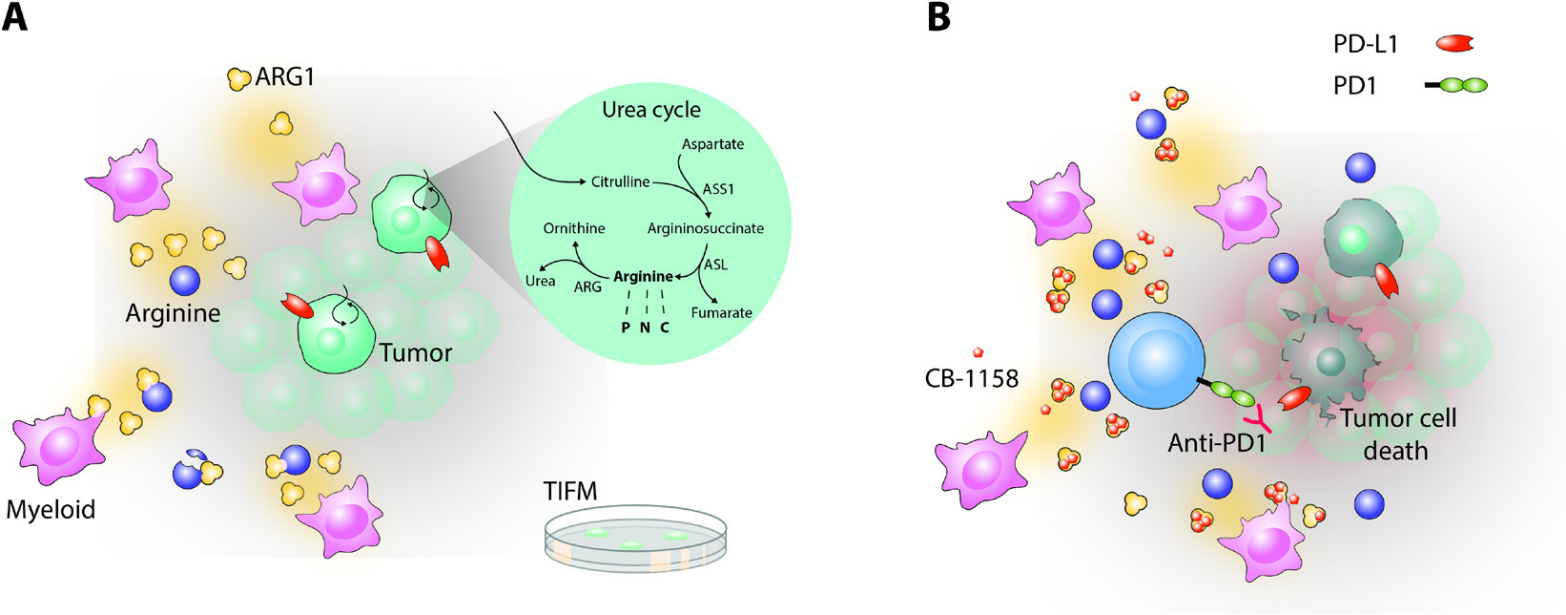
Depletion of arginine in the tumor microenvironment shapes how pancreatic cancer cells behave and respond to drug treatment in the laboratory. (**A**) The microenvironment surrounding pancreatic ductal adenocarcinoma cells (PDA, green) contains myeloid cells (pink) that secrete an enzyme called arginase 1 (ARG1, yellow trimers): this enzyme breaks down arginine (dark blue circles), which leads to depleted levels of this amino acid. The nutrient composition of the PDA microenvironment was used to formulate a new medium for pancreatic cancer cells called TIFM. PDA cells grown in TIFM, but not standard culture media, display an active urea cycle (shown in circle inset), similar to when they are grown within the body. By taking up citrulline, a key ingredient of the TIFM but not standard media, the cancer cells can short circuit the urea cycle to synthesize their own arginine. The amino acid can then be degraded into other molecules (ornithine and urea) that help to dispose of excess nitrogen, or used to synthesize metabolic products that may promote tumor growth, such as proteins (P), nitric oxide (N) and creatine (C). (**B**) Treating mice bearing PDA tumors with the arginase inhibitor CB-1158 (small orange pentagons) raises arginine levels in the tumor microenvironment and promotes infiltration of anti-tumor CD8^+^ T cells (light blue). However, many of these immune cells are ‘exhausted’ or inactive due to their programmed cell death protein (PD1, green) binding to its ligand on the surface of tumor cells (PD-L1, red). Adding a drug that blocks this interaction called Anti-PD1 (Y-shaped red protein) helps to reactivate the CD8^+^ T cells, leading to the death of PDA cancer cells and reduced tumor growth.

Image credit: Tom DiCesare (CC BY 4.0)

By closely mimicking the nutrient composition of the tumor microenvironment, TIFM allows researchers to better understand how PDA cells overcome metabolic challenges, like low levels of arginine. It also highlights metabolic vulnerabilities which would be missed when using standard media, such as the fact that cancer cells may depend on the urea cycle to synthesize the arginine they need to grow. However, TIFM does not explain why arginine is depleted inside the PDA tumor microenvironment.

Menjivar et al. (who are based at the University of Michigan, Henry Ford Pancreatic Cancer Center, Rogel Cancer Center, and University of California Irvine) were able to provide an answer to this question by building on previous work they had done on myeloid cells, a group of immune cells which support cancer progression (Zhang et al., 2017). Their 2017 study showed that early malignant cells with a specific mutation could induce the expression of the enzyme arginase 1 (ARG1) in myeloid cells surrounding them. However, whether the enzyme’s ability to breakdown arginine influences tumor growth had not been fully explored.

Menjivar et al. found that a high number of myeloid cells expressing the gene for ARG1 were present in the microenvironment of human and mice pancreatic tumors. Depleting this gene from the myeloid cells of mice resulted in higher levels of arginine, an effect known to alter the fitness of immune cells (Geiger et al., 2016). This helped to recruit anti-cancer CD8^+^ T cells to the tumor microenvironment and delayed disease progression.

However, many of these mice still developed invasive pancreatic cancer, which Menjivar et al. attribute to two factors. First, a compensatory mechanism which allows myeloid cells to produce the mitochondrial form of arginase known as ARG2, and pancreatic cells to synthetize ARG1. Second, a large portion of the CD8^+^ T cells infiltrating the PDA tumors are in fact ‘exhausted’ and not fully functioning. The compensatory expression of ARG1 and ARG2 could be counteracted by pharmacologically inhibiting all extracellular arginases; however, tumor reduction was most pronounced when the arginase inhibitor CB-1158 was combined with a drug that could reactivate exhausted CD8^+^ T cells and boost their anti-tumor role (Figure 1B).

The findings of Menjivar et al. demonstrate why arginine is depleted in the pancreatic tumor environment, and how this change in nutrient availability affects immune cells and influences tumor progression. In turn, arginine depletion formed the basis of the customized medium formulated by Apiz Saab, Dzierozynski et al., which allowed them to probe the metabolic changes associated with PDA.

Intriguingly, Apiz Saab, Dzierozynski et al. found that tumor cells sustained their urea cycle activity to produce arginine even when myeloid cells could not produce arginase anymore, and the amino acid was available in the environment. Not rapidly taking advantage of this new source of arginine may seem counterintuitive and energetically costly for the cells, but maintaining their own production may be beneficial. For example, maintaining an active urea cycle could help cancer cells dispose of the nitrogen they accumulate due to their malignant activity, or allow them to better channel arginine into cancer-promoting processes (Zaytouni et al., 2017).

In addition, not metabolically re-adapting from a nutrient-starved to a nutrient-replete state could protect cancer cells from sudden fluctuations in nutrient availability that could lead to cell cycle arrest or death. Such changes could result from growing tumors experiencing rapid alterations in their three-dimensional landscape which alter how close they are to blood vessels or other cell types that deplete the environment of specific nutrients. Consistent with this idea, PDA cells were recently shown to unconventionally rely on glutamine rather than arginine to synthetize the precursors needed to build polyamines, a class of molecules important in cancer progression. This unusual glutamine-derived pathway persisted even when arginine became widely available, suggesting that the cells had been rewired to favor this biochemical process (Lee et al., 2023).

Taken together, these two studies show how investigating the behavior of pancreatic cancer cells in response to nutrient availability can lead to a deeper understanding of the biology of an aggressive, uncooperative disease. In particular, they have unveiled metabolic challenges and vulnerabilities that could become therapeutic targets. Studying the ever-changing nutrient composition of the tumor microenvironment should prove to be a promising tool to identify the distinct metabolic features that cancer cells rely on in a range of tissues and at various stages of the disease.

## References

[bib1] Apiz Saab JJ, Dzierozynski LN, Jonker PB, AminiTabrizi R, Shah H, Menjivar RE, Scott AJ, Nwosu ZC, Zhu Z, Chen RN, Oh M, Sheehan C, Wahl DR, Pasca di Magliano M, Lyssiotis CA, Macleod KF, Weber CR, Muir A (2023). Pancreatic tumors exhibit myeloid-driven amino acid stress and upregulate arginine biosynthesis. eLife.

[bib2] Cantor JR, Abu-Remaileh M, Kanarek N, Freinkman E, Gao X, Louissaint A, Lewis CA, Sabatini DM (2017). Physiologic medium rewires cellular metabolism and reveals uric acid as an endogenous inhibitor of UMP synthase. Cell.

[bib3] Geiger R, Rieckmann JC, Wolf T, Basso C, Feng Y, Fuhrer T, Kogadeeva M, Picotti P, Meissner F, Mann M, Zamboni N, Sallusto F, Lanzavecchia A (2016). L-Arginine modulates T cell metabolism and enhances survival and anti-tumor activity. Cell.

[bib4] Hanahan D, Weinberg RA (2011). Hallmarks of cancer: the next generation. Cell.

[bib5] Lee MS, Dennis C, Naqvi I, Dailey L, Lorzadeh A, Ye G, Zaytouni T, Adler A, Hitchcock DS, Lin L, Hoffman MT, Bhuiyan AM, Barth JL, Machacek ME, Mino-Kenudson M, Dougan SK, Jadhav U, Clish CB, Kalaany NY (2023). Ornithine aminotransferase supports polyamine synthesis in pancreatic cancer. Nature.

[bib6] Martínez-Reyes I, Chandel NS (2021). Cancer metabolism: looking forward. Nature Reviews Cancer.

[bib7] Menjivar RE, Nwosu ZC, Du W, Donahue KL, Hong HS, Espinoza C, Brown K, Velez-Delgado A, Yan W, Lima F, Bischoff A, Kadiyala P, Salas-Escabillas D, Crawford HC, Bednar F, Carpenter E, Zhang Y, Halbrook CJ, Lyssiotis CA, Pasca di Magliano M (2023). Arginase 1 is a key driver of immune suppression in pancreatic cancer. eLife.

[bib8] Sullivan MR, Danai LV, Lewis CA, Chan SH, Gui DY, Kunchok T, Dennstedt EA, Vander Heiden MG, Muir A (2019). Quantification of microenvironmental metabolites in murine cancers reveals determinants of tumor nutrient availability. eLife.

[bib9] Vande Voorde J, Ackermann T, Pfetzer N, Sumpton D, Mackay G, Kalna G, Nixon C, Blyth K, Gottlieb E, Tardito S (2019). Improving the metabolic fidelity of cancer models with a physiological cell culture medium. Science Advances.

[bib10] Zaytouni T, Tsai PY, Hitchcock DS, DuBois CD, Freinkman E, Lin L, Morales-Oyarvide V, Lenehan PJ, Wolpin BM, Mino-Kenudson M, Torres EM, Stylopoulos N, Clish CB, Kalaany NY (2017). Critical role for arginase 2 in obesity-associated pancreatic cancer. Nature Communications.

[bib11] Zhang Y, Yan W, Mathew E, Kane KT, Brannon A, Adoumie M, Vinta A, Crawford HC, Pasca di Magliano M (2017). Epithelial-myeloid cell crosstalk regulates acinar cell plasticity and pancreatic remodeling in mice. eLife.

